# Risk of Lower Extremity Ligamentous Injury Following Concussion Diagnosis: A TriNetX Database Study

**DOI:** 10.7759/cureus.35908

**Published:** 2023-03-08

**Authors:** Morgan Birrell, Andrea H Johnson, Jane C Brennan, Benjamin M Petre, Justin J Turcotte, Daniel E Redziniak

**Affiliations:** 1 Internal Medicine, Anne Arundel Medical Center, Annapolis, USA; 2 Orthopedic Research, Anne Arundel Medical Center, Annapolis, USA; 3 Orthopedic Surgery, Anne Arundel Medical Center, Annapolis, USA

**Keywords:** neuromuscular evaluation, concussion, lower extremity injury, ankle sprain, knee ligament injury

## Abstract

Background

Concussion is one of the most frequently reported sports-related injuries in the United States; there is evidence that residual deficits in neurocognition may increase the risk of lower extremity musculoskeletal injury after concussion in high school, college, and professional athletes. The purpose of this study is to identify whether similar trends are identified in community-based populations.

Methods

The TriNetX Research Network database was queried for patients 10-60 years old who experienced an ambulatory or emergency visit from 2018-2020. Cohorts were defined by patients seen for concussion and patients seen for other reasons. These cohorts were then propensity score matched based on significant differences in demographics; after matching, each cohort included 97,708 patients. The propensity score-matched cohorts were then evaluated to identify patients who experienced subsequent lower extremity ligamentous injury within 12 months.

Results

Patients with a history of concussion were more likely to experience posterior cruciate ligament (PCL) sprain (0.04% vs. 0.02%, risk ratio (RR)=1.79, p=.039), medial collateral ligament (MCL) sprain (0.18% vs. 0.08%, RR=2.355, p<.001), lateral collateral ligament (LCL) sprain (0.05% vs. 0.02%, RR=2.202, p=.003) and ankle sprain (1.05% vs. 0.47%, RR=2.265, p<.001).

Conclusion

Patients diagnosed with concussion were more likely to experience a lower extremity ligamentous injury when compared with patients who did not have concussion. Patients should be counseled regarding this increased risk and additional neuromuscular evaluation and injury prevention education may be indicated following concussion diagnosis.

## Introduction

Concussion is one of the most frequent sports-related injuries in the United States (US) and according to the CDC, about 3.8 million people sustain a concussion each year related to sports and recreation in the US [1]. A well-studied association in athletes is the predisposition for further injury, especially lower extremity injury, in a period following concussion. The follow-up period ranges from days to years depending on the study, but follow-up of more than 10 years has also been examined. Importantly, the increased risk of injury extends well beyond the period when clinical signs and symptoms resolve, suggesting that athletes may be unaware that they are at increased risk [2]. Taken together, these data often aim to inform safe “return to play” recommendations and may also serve to educate athletes and coaches on the increased risk of injury in the period following concussion.

Sports medicine physicians and other general practitioners in the community setting are likely to encounter patients with concussions and need to provide recommendations for when it is safe to return to recreational sports, day-to-day activities, and work. To date, there has been limited evaluation of whether the association between concussion and subsequent lower extremity injury exists in the community-based population setting. We aim to examine retrospective data from a global research network (TriNetX) for patients who have sustained concussions to determine if this trend is relevant in the community-based practice setting. If so, this may inform recommendations for return to work and day-to-day activities for these patients. In addition, it suggests another important facet of patient education following concussion as patients can be empowered to protect themselves from future injury with the knowledge that they may be at increased risk.

This article was previously presented as a poster at the 2022 American College of Surgeons Clinical Congress on October 18, 2022. This article has also been accepted for poster presentation at the 2023 American Academy of Orthopedic Surgeons Annual Meeting on March 9, 2023.

## Materials and methods

The TriNetX Research Network database was queried on January 11, 2022. Patient cohorts and outcome measures were defined using the International Classification for Disease, 10th Edition (ICD-10) diagnosis codes. All patients aged 10-60 years old who experienced an ambulatory or emergency visit between January 1, 2018, and December 31, 2020, were included and divided into two cohorts according to the presence or absence of a concussion diagnosis at the initial visit. Each cohort was then queried to identify a subsequent lower extremity injury diagnosed within 12 months of the initial visit. A total of 16,695,899 (concussion n=97,708; no concussion n=16,598,191) patients were included in the study.

The two cohorts were then propensity score matched on age, gender, and diagnosis of overweight or obese. The cohorts were then examined for the presence of an anterior cruciate ligament (ACL) sprain (ICD-10 code S83.51), posterior cruciate ligament (PCL) sprain (ICD-10 code S83.52), medial collateral ligament (MCL) sprain (ICD-10 code S83.41), lateral collateral ligament (LCL) sprain (ICD-10 code S83.42), and ankle sprain (ICD-10 code S93.4). Categorical outcomes were compared between cohorts using z-tests and the risk ratio (RR) and 95% confidence interval were calculated. Continuous outcome measures were compared using two-sided independent sample t-tests. Statistical significance was assessed at α=.05. All statistical analysis was performed within the TriNetX platform.

About TriNetX

TriNetX is a global research network that includes data from more than 170 healthcare organizations across 30 countries and over 400 million patients [3]. Variables captured include demographics, medications, lab values, diagnoses (mapped to ICD-10 coding), and procedures (Current Procedural Terminology (CPT®)). Health Insurance Portability and Accountability Act (HIPAA) compliant electronic health record data were collected from participating healthcare organizations that submit structured and unstructured data elements.

Ethical Considerations

TriNetX is a federated network and as such, this study received a waiver from Western Institutional Review Board as the only data received included aggregated counts and statistical summaries of de-identified information. No protected health information is exchanged in retrospective analyses.

## Results

Prior to propensity score matching, patients with a concussion were significantly younger, less likely to be female, and less likely to be overweight or obese (all p<.001). After propensity score matching, 97,708 patients were included in each group; all demographics were equally matched (all p=1.000) (Table 1).

**Table 1:** Patient Demographics Before and After Propensity Score Matching

	Before Matching			After Matching		
	Concussion ( N=97,708)	No Concussion (N=16,598,191)	P-value	Concussion (N=97,708)	No Concussion (N-97,708)	P-value
Age in Years	26.2 ± 13.7	33.6 ± 14.5	< .001	26.2 ± 13.7	26.2 ± 13.7	1.000
Female Sex	50,477 (51.7)	9,493,791 (57.2)	< .001	50,477 (51.7)	50,477 (51.7)	1.000
Overweight or Obese	5058 (5.2)	932,153 (5.6)	< .001	5058 (5.2)	5058 (5.2)	1.000

Patients with a history of concussion were more likely to experience a PCL sprain (0.04% vs. 0.02%, RR=1.79, 95%CI: 1.021-3.138, p=.039), MCL sprain (0.18% vs. 0.08%, RR=2.355, 95% CI 1.797-3.085, p<.001), LCL sprain (0.05% vs. 0.02%, RR=2.202, 95%CI 1.298-3.736, p=.003) and ankle sprain (1.05% vs. 0.47%, RR=2.265, 95%CI 2.026-2.534, p<.001). There was no significant difference in the rate of ACL sprain between groups (Table 2).

**Table 2:** Incidence of Lower Extremity Soft Tissue Injury within One Year of Concussion ACL, anterior cruciate ligament; PCL, posterior cruciate ligament; MCL, medial collateral ligament; LCL, lateral collateral ligament

	Concussion, N (%)	No Concussion, N (%)	Risk Ratio (Concussion: No Concussion)	95% CI	P-value
ACL Sprain/Tear	153 (0.16)	148 (0.15)	1.035	0.826-1.297	0.766
PCL Sprain/Tear	34 (0.04)	19 (0.02)	1.79	1.021-3.138	0.039
MCL Sprain/Tear	176 (0.18)	75 (0.08)	2.355	1.797-3.085	< .001
LCL Sprain/Tear	44 (0.05)	20 (0.02)	2.202	1.298-3.736	0.003
Ankle Sprain	972 (1.05)	444 (0.47)	2.265	2.026-2.534	< .001

## Discussion

It has been well established that for individuals sustaining a concussion, there is an increased risk of subsequent lower extremity injury in the months following the index head injury [4-7]. However, the available literature assessing this relationship, as well as the underlying neuromechanical mechanisms, has focused primarily on high school, college, and professional athletes. For example, Lynall et al. found that college athletes with prior concussions, when matched with non-concussed control athletes, were 1.64 times more likely to experience a lower extremity injury [8]. In their prospective study of elite European football players, Nördstrom et al. found that a history of concussion results in an increased risk of subsequent injury compared to controls in a time-dependent fashion, with the greatest risk at 6-12 months post concussion; lower extremity injury accounted for 83% of their data set [9]. The relationship may also be dose-dependent, allowing Harada et al. to show that lower extremity injury was higher in division I athletes with multiple concussions compared to those with one or no concussions [10].

Here, we reviewed data from nearly 100,000 concussion patients in the general population to determine if this association is relevant in the community setting. In congruence with the athlete population, community-based patients with a history of concussion in the last 12 months displayed an increased risk of lower extremity ligamentous injury when compared to their propensity-matched controls. Specifically, a prior concussion increases the risk of subsequent LCL, MCL, PCL, or ankle sprain compared to those who have not had a concussion; the association with ACL sprain was not significant in our cohort. These findings suggest that more intervention may be warranted when concussion patients are encountered in the community-based practice setting. While patient education is essential, a more comprehensive injury prevention effort is further supported by understanding the neuromechanical processes that may predispose patients to injury after a concussion.

Sequelae of a concussion that may pre-dispose to injury are also largely studied in the athlete population and are usually evaluated in the context of “safe return to play” recommendations. Importantly, the time to full physiologic recovery is thought to extend beyond the time at which salient clinical signs and symptoms of concussion resolve [2]. This is at odds with current recommendations to use clinical assessment to direct a graduated return to play. For example, De Beaumont et al. found that ongoing deficits in postural control were displayed in collegiate football players more than nine months following concussion [11]. However, concussion-associated symptoms may subside as early as 7-10 days following the initial injury [9]. Other such post-concussion changes include decreased postural stability, increased reaction time, changes in gait pattern, decreased maximum strength, lower extremity stiffness, and altered gait strategy [2,5,12-14].

The impacts of these physiologic changes on injury risk are also well documented. Hrysomallis found that poor postural stability was associated with an increased risk of ankle injury [15]. Furthermore, deficits in reaction time have also been linked to lower extremity injury [4]. These findings, coupled with the persistence of physiologic changes beyond the period of active clinical concussion symptoms, offer a plausible biophysical mechanism for increased lower extremity injury remote from prior concussion. Additionally, and not to be minimized in this setting are the psychological sequelae of injury, such as stress, sleep deprivation, and attention deficits, which also appear to impact the risk of injury in various athlete populations [2,7,16].

Our findings highlight the importance of patient education following a concussion, with a specific focus on the hidden deficits that may predispose them to a second injury. Patients can be empowered to protect themselves from further injury if given ample description of the factors that place them at increased risk. In addition, with attention to the physiologic changes such as gait, strength, and postural stability, there may also be value in more structured neuromuscular evaluations to inform return to activity recommendations. This is of particular importance in the community-practice setting where work hours, workplace injury risk, and financial productivity can be affected by inappropriate liberal or conservative recommendations.

There are some limitations to our study, namely the use of a conglomerate retrospective database source. This impairs the amount of additional information that can be obtained for each patient. For example, insight into the cause of secondary lower extremity injury, the presence of other concomitant injuries, and the degree of ligament injury are absent. While not essential, this information would prove useful in counseling patients about the risks of injury and prevention measures in the post-concussion period.

Similarly, using only the ICD-10 code as the identifier for patients with concussion also precludes significant granularity with regard to the severity of the initial head injury. It may be that a clinically relevant risk of lower extremity injury appears only above a certain degree of initial head injury severity. This may be more important in counseling patients about return to work or school when extended absence can have financial impacts. While the default should be to err on the side of patient safety, an accurate understanding of post-concussion lower extremity injury risk will allow for optimal return-to-activity recommendations.

## Conclusions

There are ample extant data indicating that athletes at the high school, college, and professional levels are at an increased risk for lower extremity ligamentous injury following a recent concussion. While it is reasonable to suspect that the same post-concussion risk of injury extends to the general population, these outcomes have not yet been scrutinized in a community-practice setting. In our retrospective review of nearly 100,000 concussion patients, we found that suffering a concussion leads to an increased risk of subsequent lower extremity ligamentous injury within the next year, as compared to propensity-matched controls. With this in mind, it seems prudent to educate concussion patients on these risks, as well as means of injury prevention. In some patients, a more focused evaluation of their balance, strength, and gait may aid in directed injury prevention.

Further research may more closely examine the inciting event for lower extremity ligamentous injury in the post-concussion period. Identifying day-to-day activities that place patients at increased risk for injury may again prove useful for patient counseling, and better direct when patients can return to their pre-concussion level of work or activity. Exploring the differential impact of initial concussion severity may be another avenue that warrants further evaluation.
